# Recombinant LPG3 Protein From *Leishmania chagasi* as an Antigen for the Diagnosis of Canine Visceral Leishmaniasis

**DOI:** 10.1111/tmi.70151

**Published:** 2026-04-28

**Authors:** Thaís Viana Fialho Martins, Anna Cláudia Alves de Souza, Bianca Meireles Miranda, Ronny Francisco de Souza, Daniel Silva Sena Bastos, Neverton José Silva Ferreira, Tiago Antônio de Oliveira Mendes, Celeste da Silva Freitas de Souza, George Luiz Lins Machado‐Coelho, Maria Terezinha Bahia, Leandro Licursi de Oliveira, Juliana Lopes Rangel Fietto, Eduardo de Almeida Marques‐da‐Silva

**Affiliations:** ^1^ Department of General Biology Federal University of Viçosa Viçosa Minas Gerais Brazil; ^2^ Department of Biochemistry and Molecular Biology Federal University of Viçosa Viçosa Minas Gerais Brazil; ^3^ University Center of Caratinga‐UNEC Educational Foundation of Caratinga‐FUNEC Caratinga Minas Gerais Brazil; ^4^ Oswaldo Cruz Institute Oswaldo Cruz Foundation Rio de Janeiro Rio de Janeiro Brazil; ^5^ Research Center in Biologic Sciences – NUPEB Federal University of Ouro Preto Ouro Preto Minas Gerais Brazil

**Keywords:** canine visceral leishmaniasis, diagnosis, heparin‐binding protein, *Leishmania chagasi*, recombinant lipophosphoglycan 3 protein, zoonosis

## Abstract

Visceral leishmaniasis (VL) is a human neglected tropical disease in which dogs participate as reservoirs of the etiological agent *Leishmania chagasi*. The identification of infected dogs is important to the control of VL, and recombinant proteins are strong antigen candidates for canine visceral leishmaniasis (CVL) serodiagnosis. In this work, we evaluated the enzyme‐linked immunosorbent assay (ELISA) using the *L. chagasi* LPG3 recombinant protein (rLPG3) as an antigen for the CVL diagnosis. The tests were performed on 242 serum samples and showed a remarkable 95.71% sensitivity and 87.50% specificity, with cross‐reactivity observed in some samples positive for *Ehrlichia canis* and *Trypanosoma cruzi*. The performance of the rLPG3‐ELISA test was similar to the performance of IFI‐CVL and IFI‐CVL plus ELISA‐CVL Bio‐Manguinhos tests, widely used in the diagnosis of CVL in Brazil, and presented better sensitivity results compared to those obtained in rapid tests available for CVL using other antigenic proteins. Further refinement is necessary to minimize the cross‐reactivity and enhance the specificity of the test. Studies to discriminate cross‐reactive epitopes of rLPG3, particularly those shared with *E. canis* protein sequences, may help improve diagnostic accuracy by excluding these epitopes from the detection reaction.

## 1 Introduction

Leishmaniasis is a largely neglected tropical disease caused by different species of the genus *Leishmania*, which are obligatory intracellular parasites transmitted to vertebrate hosts by the female sandfly bite during its blood meals. The overall incidence of this disease is 600,000 to 1 million new cases of cutaneous leishmaniasis (CL), and 50,000–90,000 new cases of visceral leishmaniasis (VL) annually, at a global level, with just 25%–45% being reported to the World Health Organization [1]. In Latin America alone in 2022, a total of 1833 cases of VL were reported, with 91.87% occurring exclusively in Brazil [2].

VL is a potentially fatal disease that represents a serious public health issue, with major incidence in Central and South America, Mediterranean countries, India, Bangladesh, and a few African countries (Sudan, South Sudan and Ethiopia). The disease is responsible for around 20,000–40,000 deaths per year worldwide, including cases of *Leishmania donovani* and *Leishmania infantum* infections in the Old World, and *Leishmania chagasi* in the New World [3]. A few years ago, the disease was observed to spread to non‐endemic areas, including Canada, the United States, Italy and Germany [4, 5].

Dogs are the domestic reservoirs of the New World *Leishmania*. They are epidemiologically important to the urbanization process of leishmaniasis and represent a zoonotic risk to human populations in the endemic areas. Dogs may suffer from a severe disease, canine visceral leishmaniasis (CVL), characterized by a wide range of clinical signs that manifest a few months after exposure to the parasite (skin lesions, weight loss, and generalized lymphadenomegaly), with the risk of death [6, 7].

The early and correct diagnosis of leishmaniasis in both dogs and humans is a strategy used to control the spread and mortality resulting from VL [8]. There are approximately 20–30 million dogs at risk in the geographical regions of Brazil, where CVL is endemic. The seroprevalence of the symptomatic and asymptomatic dogs is between 3.4% and 40%, revealing the potential for surveillance of canine infection as a transmission marker [9]. Among the strategies used in the control of infectious diseases, immunodiagnosis is crucial to demonstrate the infection and plays various roles in studies on disease monitoring and as a tool to monitor the control strategies and assist in surveillance during the prevention and control campaigns [10].

Diagnostic methods that rely on the visualization of parasites in bone marrow smears and aspirates from the spleen, liver and lymph nodes are highly invasive, time‐consuming and unsuitable for large‐scale epidemiological surveillance. As a result, conventional diagnostic approaches for CVL primarily focus on serological assays, such as direct agglutination, indirect immunofluorescence and enzyme‐linked immunosorbent assay (ELISA). However, these methods frequently produce false‐positive results, particularly in dogs co‐infected with other pathogens, due to antigenic cross‐reactivity [11, 12, 13]. Furthermore, false‐negative results are prevalent with conventional methods [14], underscoring the need for more sensitive diagnostic techniques.

*Leishmania* recombinant proteins have been used as a target for serodiagnosis in the pursuit of higher sensitivity and specificity of the VL immunodiagnostic tests. In this scenario, several proteins showed promising results for such use, such as CatL [15], rK39 [16], LACK, KMPII, TRYP [17, 18], rLic‐NTPDase 2 [19], rSMP3 [20], heat shock proteins [21], rcyspep [22] and salivary recombinant proteins rSP03B and rSP01 [23]. Importantly, studies have consistently demonstrated that recombinant proteins provide superior diagnostic accuracy compared to crude antigen‐based tests, further reinforcing their potential as more reliable and effective tools for serological diagnosis [20, 24, 25].

The LPG3 protein from *L. chagasi* was studied by our research group, where we identified its ability to bind to heparin. Furthermore, our team successfully evaluated LPG3 as a vaccine candidate, demonstrating its efficacy in inducing protective immune responses using both its native and recombinant forms [26, 27, 28, 29]. The LPG3 is predicted to have ATP‐binding and protein‐folding functions through its HATPase_C and HSP90 domains. Our in situ assays demonstrated that rLPG3 hydrolyses ATP and that heparin competes for its ATP‐binding site, inhibiting ATPase activity [29]. This suggests that heparin regulates ATP metabolism and influences the uptake of extracellular metabolites for purine nucleotide synthesis. Additionally, LPG3 plays a crucial role in macrophage infection by promastigotes, with heparin blocking impairing this process [26]. Moreover, the phylogenetic analysis of its sequence indicated a difference from the homologous sequence of *Trypanosoma cruzi*, thus reducing the potential for cross‐reactivity with this parasite.

In this work, the recombinant LPG3 protein (rLPG3) was successfully expressed and purified for use as an antigen in an ELISA assay to evaluate its potential for the serological diagnosis of CVL. Beyond its application in CVL, we also assessed its cross‐reactivity with sera from dogs affected by other infectious diseases, such as ehrlichiosis and Chagas disease, to determine its specificity and broader diagnostic validity.

## 2 Methods

### 2.1 Recombinant Protein Expression and Purification

The DNA sequence encoding LPG3 was synthetically produced and cloned by GenOne (Rio de Janeiro, RJ, Brazil). The gene was initially inserted into the pUC19 vector using NdeI and XhoI restriction sites and subsequently subcloned into the corresponding sites of the pET‐28a^(+)^ expression vector (Novagen, San Diego, CA, USA), which provides an N‐terminal His‐tag fusion. The recombinant plasmid was propagated in *Escherichia coli* DH5α cells.

For protein expression, *E. coli* Rosetta (DE3) cells transformed with the pET‐28 construct were cultured in Luria–Bertani (LB) medium supplemented with kanamycin (50 μg mL^−1^) and chloramphenicol (34 μg mL^−1^) at 37°C with shaking (200 rpm). When cultures reached mid‐log phase (OD600 ≈ 0.6–0.8), protein expression was induced with 1.0 mmol mL^−1^ isopropyl β‐d‐1‐thiogalactopyranoside (IPTG) for 4 h at 37°C. Bacterial cells were harvested and lysed by sonication in 10 mmol mL^−1^ sodium phosphate and 150 mmol mL^−1^ sodium chloride buffer (pH 7.0; buffer A). The soluble protein fraction was collected and purified by Ni‐NTA affinity chromatography (Qiagen, Hilden, Germany) pre‐equilibrated with buffer A. Bound proteins were eluted using buffer A supplemented with 500 mmol mL^−1^ imidazole (buffer B). Eluted fractions were analysed by 10% SDS‐PAGE and further purified by heparin–agarose affinity chromatography using an ÄKTA purifier system (GE Healthcare Life Sciences, Freiburg, Germany), equilibrated with buffer A. The retained proteins were eluted with buffer A containing 2 mol L^−1^ NaCl. The eluate was subsequently subjected to size‐exclusion chromatography on a Superdex 200 10/300 GL column (GE Healthcare Life Sciences). Protein purity was confirmed by Coomassie‐stained 12% SDS‐PAGE, and protein concentration was determined using the Lowry method.

### 2.2 Serum Samples

A total of 242 serum samples from dogs were used in the rLPG3‐ELISA assay. The efficacy of the test, cross reaction (Chagas disease and ehrlichiosis) and comparison of LPG3‐ELISA with standard diagnostic kits were evaluated. The negative sera for *L. chagasi* infection (30 samples) were acquired from dogs maintained at the kennel of the Federal University of Ouro Preto (UFOP) under controlled conditions. These animals presented negative results in the parasitological and/or polymerase chain reaction (PCR) assays. Positive sera for *L. chagasi* infection were acquired from different endemic regions: 48 samples from Caratinga, MG, Brazil, and 31 samples from Governador Valadares, MG, Brazil. The positive sera were kindly provided by Fundação Oswaldo Cruz (Fiocruz, Rio de Janeiro, RJ), head office, Belo Horizonte, MG. Serum samples were considered positive for CVL infection when confirmed by parasitological and/or PCR tests.

To evaluate the effectiveness of the proposed test, we compared the diagnosis using LPG3‐ELISA in 2 groups of sera positive for *L. chagasi*. The first one was assessed by indirect immunofluorescence (IFI) and the second one through IFI plus the standard ELISA kit Bio‐Manguinhos (Instituto de Tecnologia em Imunobiológicos, Rio de Janeiro, RJ, Brazil)—43 and 41 samples, respectively. Fiocruz also provided these groups of sera.

To evaluate cross‐reactivity, sera from dogs experimentally infected with *T. cruzi* (39 samples) and 10 sera samples from *Ehrlichia canis* ‐positive dogs were used. The positive *T. cruzi* serum samples are part of the serum library of UFOP and were provided by Professor Maria Terezinha Bahia. The Chagas disease‐positive animals were bred and maintained at the UFOP kennel under controlled conditions. The infection was confirmed through direct parasitological examination and/or polymerase chain reaction (PCR). The *E. canis* ‐positive serum samples were provided by Professor Celeste da Silva Freitas de Souza from the Laboratory of Immunomodulation and Protozoology at Fiocruz, with prior diagnosis using the SNAP 4Dx Plus Test—Canine *Leishmania* Antibody Test (IDEXX Laboratories Inc., Westbrook, Maine, USA).

### 2.3 Ethics Statement

The collection of serum samples was carried out in strict accordance with the Brazilian laws on animal experimentation. The use of the negative sera for *L. chagasi* infection obtained from animals maintained at the kennel of the UFOP and sera from dogs experimentally infected with *T. cruzi* was approved by the Ethics Committee of Animal Research of the Federal University of Ouro Preto (numbers: 2012/18 and 2015/53, respectively). The use of the samples from ehrlichiosis‐positive dogs was approved by the Animal Use Committee of the Oswaldo Cruz Foundation (authorization: LW‐33/11). The other serum samples were obtained from the Monitoring and Control Program of Visceral Leishmaniasis (VLMCP), and, according to Brazilian law, in this specific situation, there is no need for registration by an animal experimentation ethics committee. Sera were not tested blind to infection status.

### 2.4 ELISA

Microplates with 96 wells were coated with rLPG3 (0.5 μg/well) in coating buffer (0.1 mol L^−1^ Na_2_CO_3_/NaHCO_3_, pH 9.6) at 4°C for 18 h. Plates were washed four times with PBS 0.05% Tween 20 and blocked with PBS 3% BSA for 1 h at room temperature. In a preliminary optimization analysis, the antigens were tested at concentrations of 0.5, 1, 2, 5 and 10 μg/well, and the sera at dilutions of 1:40, 1:100, 1:200, 1:300, 1:400 and 1:800. The results obtained indicated the use of 0.5 μg of antigen per well and a 1:40 dilution of the serum samples.

Sera from dogs (diluted 1:40 in PBS/1% BSA) were added and incubated for 1 h at room temperature. After four washes with PBS/0.05% Tween 20, 1:5000 diluted rabbit peroxidase‐conjugated anti‐dog IgG antibody (Sigma Aldrich, St. Louis, Missouri, USA) was added, and the material was incubated for 1 h at room temperature. The plates were washed four times with PBS/0.05% Tween 20, and 100 μL of 0.5 mg mL^−1^ 2,2′‐Azino‐bis (3‐ethylbenzothiazoline‐6‐sulfonic acid) diammonium salt (ABTS) substrate (Sigma‐Aldrich) in 0.1 mol L^−1^ citrate buffer pH 5.0 containing hydrogen peroxide were added. The plates were incubated for 30 min at room temperature, and the reaction was stopped with 100 μL 1% SDS. The absorbance was measured at 405 nm. Each serum sample was evaluated in triplicate.

### 2.5 Statistical Analysis

The receiver operating characteristic (ROC) curve was constructed using GraphPad Prism version 6.0 (GraphPad Software, San Diego, CA, USA) by plotting sensitivity against (1 − specificity) across all possible cutoff values. The area under the ROC curve (AUC) was used to evaluate the overall discriminative performance of the test. The optimal cutoff was determined using the Youden index (*J* = sensitivity + specificity − 1). Sensitivity and specificity were then calculated from a 2 × 2 contingency table based on observed classifications at the selected cutoff, using parasitological and/or PCR results as the reference standard. Indeterminate results were excluded from the analysis. Positive and negative likelihood ratios (LR+ and LR−) were calculated from sensitivity and specificity at the predefined cutoff, with LR+ defined as sensitivity/(1 − specificity) and LR− as (1 − sensitivity)/specificity.

The lower limit of positivity (cut‐off) for rLPG3‐ELISA was determined as the mean of the absorbance of 30 randomly selected negative control sera, plus two standard deviations. A grey zone range of the ±10% value of cut‐off was used to indicate the values whose absorbance did not provide conclusive information. Absorbances in the grey zone were considered indeterminate. The ELISA performance was assessed using a dichotomous approach and compared in terms of sensitivity, specificity and accuracy [30]. Confidence intervals were employed with a confidence level of 95%.

McNemar's test [31] was calculated to compare paired proportions using Quickcalcs, GraphPad (https://www.graphpad.com/quickcalcs). In the data presented using a box‐and‐whisker plot, the box extends from the 25th to the 75th percentiles. The line in the middle of the box is plotted at the mean, and the whiskers represent the highest and lowest points. Data normality was assessed using the D'Agostino–Pearson test. As the data did not follow a normal distribution, comparisons between groups were performed using the Mann–Whitney *U* test. Differences were considered statistically significant when *p*‐values were < 0.05.

## 3 Results

### 3.1 Expression and Purification of *L. chagasi* rLPG3 Antigen

The rLPG3 protein was expressed, and the purification yield was estimated to be between 14 and 16 mg L^−1^, while the molecular mass was estimated to be around 86 kDa [29]. These data were confirmed by a highly sensitive capillary electrophoresis technique coupled with fluorescence detection, with a 94.7% degree of purity (Figure S1). The purified antigen was then tested for the immunodiagnosis of *L. chagasi* infection using the ELISA method.

### 3.2 Performance of rLPG3 Antigen in the Immuno‐Diagnostic Test for CVL

**FIGURE 1:**
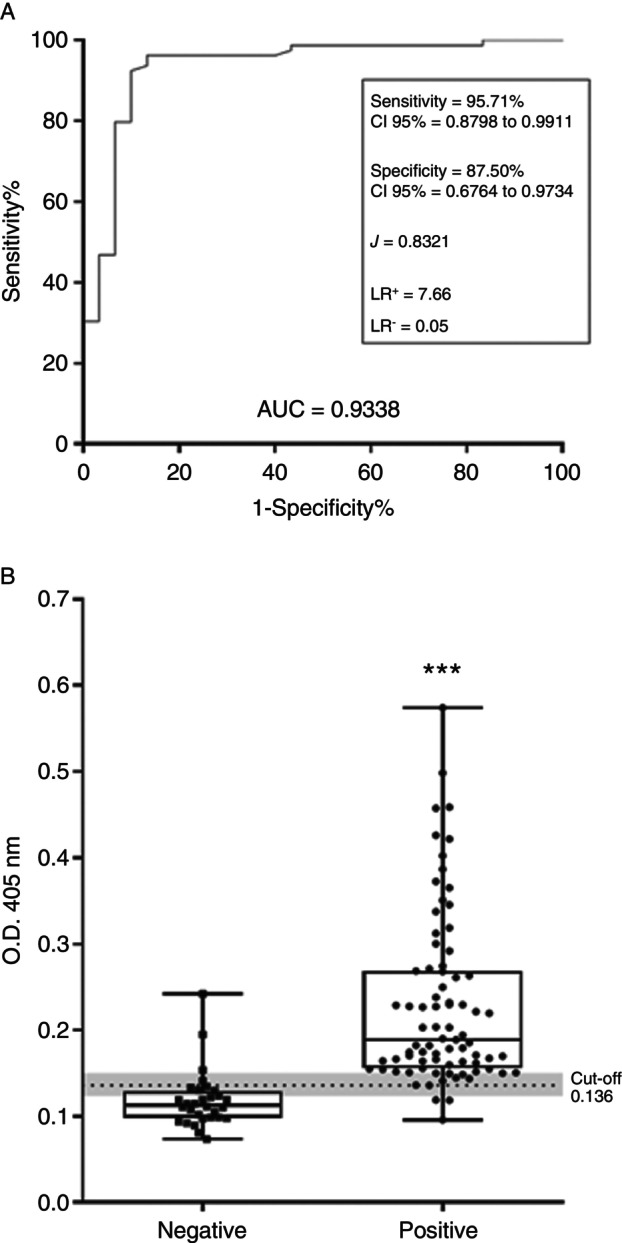
ROC curve and box‐and‐whisker plot obtained from rLPG3‐ELISA test. (A) ROC curve: Distribution of the 30 negative and 79 positives to *L. chagasi* infection serum samples; (B) *L. chagasi* infection negative and positive serum samples previously tested by parasitological and/or PCR and tested by rLPG‐ELISA. The dashed line represents the cut‐off of the test, and the grey zone is the indeterminate area. The box was plotted at the median, and the bars represent 25% and 75% Percentiles. (***) Statistical difference between groups (*p* < 0.001, Mann–Whitney *U* test); serum dilution 1:40. AUC, area under the curve; CI, confidence interval.

The rLPG3 was evaluated for the serodiagnosis of *L. chagasi* infection as the ELISA plate‐coating antigen. Prior assays demonstrated that 0.5 μg was the best quantity of protein to be used in each well, with a serum dilution of 1:40. These values provided better discrimination of the optical density values between the evaluated samples (data not shown). Based on the rLPG3‐ELISA results, a ROC curve was constructed. The area under the curve (AUC) obtained was 0.9338 (95% CI, 0.8736–0.9940), which indicates 93.38% of the overall ability of the test to correctly detect the presence of antibodies against *L. chagasi* in the samples. The sensitivity was 95.71%, and the specificity was 87.50%, with a Youden index of 0.8321 (Figure 1A). The positive likelihood ratio (LR+) was 7.66, and the negative likelihood ratio (LR−) was 0.05 (Figure 1A).

The cut‐off value was calculated by the mean plus two standard deviations of the absorbances of 10 negative control sera randomly chosen from the 30 negative control sera, resulting in the value of 0.136, with a grey zone representing ±10% of this value (Figure 1B).

**FIGURE 2:**
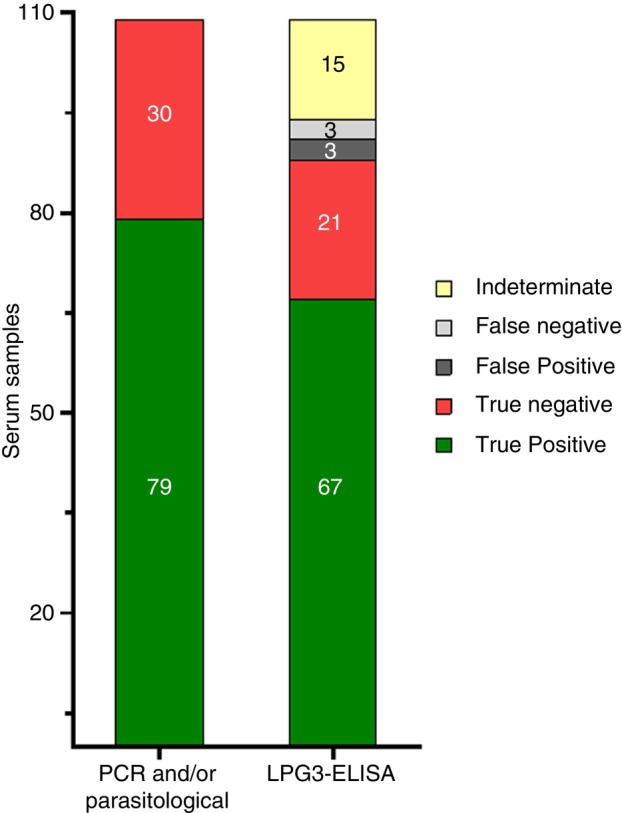
Diagnosis performance of rLPG3‐ELISA using true positive (*n* = 79) and negative (*n* = 30) sera for *L. chagasi*. The variation between the tests in terms of false negatives and false positives was non‐significant (McNemar = 0.00, *p* = 0.6831). The indeterminates were excluded from the analysis.

Among the negative control sera, 21 samples were negative (true‐negative), and three were positive (false‐positive) to the *L. chagasi* infection in the rLPG3‐ELISA. Among the positive control sera, 67 were positive in the rLPG3‐ELISA (true‐positive) and 3 were negative (false‐negative). Fifteen serum samples (13.7% of the total number of samples) fell within the grey range and, therefore, presented indeterminate results (Figures 1B and 2). According to the D'Agostino‐Pearson test, the absorbance distributions were not normal for both groups and according to the Mann–Whitney *U* test, the statistical difference between the groups was significant (*p* < 0.05), which demonstrates that the rLPG3‐ELISA can differentiate between the *L. chagasi* infection‐negative and positive sera.

The diagnosis performance evaluated by McNemar's test indicated that the difference between the rLPG3‐ELISA results and the control tests was considered statistically insignificant. The accuracy of the test was 80.7%, as 15 samples were considered indeterminate (grey zone—Figure 2).

### 3.3 Evaluating the Cross‐Reaction in rLPG3‐ELISA With *Trypanosoma cruzi* and *Ehrlichia canis* Positive Sera

**FIGURE 3:**
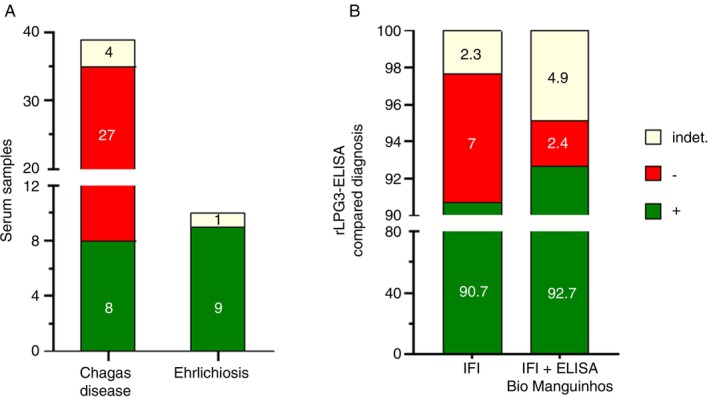
Evaluation of rLPG3‐ELISA performance. (A) Cross‐reaction analysis using 39 sera positive for anti‐ *T. cruzi* and 10 sera positive for anti‐ *E. canis* antibodies. (B) Comparison performed against conventional serological tests: Data are expressed in percentages. McNemar *p* = 0.2482, non‐significant for IFI, and *p* = 1.0 for IFI‐CVL and ELISA‐CVL. The rLPG3‐ELISA showed the same performance as indirect immunofluorescence (IFI) and the ELISA kit Bio‐Manguinhos.

The cross‐reaction of the rLPG3‐ELISA using sera from dogs with other parasitic infections was measured using 39 serum samples positive for *T. cruzi* and 10 positive for *E. canis* infections, all non‐reactive for *L. chagasi* infection. The cross‐reactivity to *T. cruzi* infection was observed in 8 samples (absorbances above the cut‐off value), which represents 20.51% of false positives. Four samples were indeterminate and 27 were negative for the *L. chagasi* infection (Figure 3A). For ehrlichiosis, 9 samples showed higher values than the cut‐off, and 1 sample was indeterminate, resulting in a 90% cross‐reactivity (Figure 3A).

### 3.4 rLPG3 Performance Comparison With Indirect Immunofluorescence or Bio Manguinhos ELISA Kit

A comparison was made between the performance of the rLPG3‐ELISA and that of conventional serological tests. As parasitological or molecular tests were not provided to confirm the results of the positive samples, these samples were classified as non‐standard samples. First, the IFI‐CVL was confronted with rLPG3‐ELISA (Figure 3B). The test was done using 43 IFI‐CVL positive samples, resulting in 39 samples (90.7%) giving positive concordant results; 3 samples (7%) were discordant (non‐reactive using the rLPG3‐ELISA, but reactive using IFI‐CVL), and 1 sample (2.3%) was classified as indeterminate. The agreement rate between these tests was 90.6%.

Among the samples diagnosed as positive by both the IFI‐CVL and ELISA‐CVL Bio‐Manguinhos, 1 (2.4%) was classified as negative by rLPG3‐ELISA, 2 (4.9%) were indeterminate and 38 (92.7%) were positive (Figure 3B). The agreement rate between these tests was 92.6%.

## 4 Discussion

The proteins associated with the infection and survival of the parasite in the intracellular environment are an important target for immunodiagnostic tests. In this context, rLPG3 is a potential target because it is present in both the cytoplasm and surface of *L. chagasi* cells [26, 32], and it participates in the infectious process in mammalian cells [26].

We expressed the rLPG3 protein in a heterologous system, producing a highly purified sample suitable for immunodiagnostic application via ELISA for the serological diagnosis of CVL. Recombinant protein antigens can be standardized and produced on a large scale due to the high yield and low cost involved in generating pure samples. Moreover, these antigens are safe, as their production does not require the maintenance and processing of live parasites [33, 34]. Additionally, soluble *Leishmania* antigens (SLA) are the parasitic proteins most employed in the diagnosis of leishmaniasis using ELISA. Although SLA‐based techniques demonstrate high sensitivity, they exhibit low specificity, leading to false‐positive diagnoses due to cross‐reactions with antibodies from *T. cruzi* ‐infected animals [18]. Consequently, ongoing efforts aim to identify new antigens suitable for the accurate diagnosis of leishmaniasis.

In the present study, we evaluated the performance of the recombinant LPG3 antigen from *L. chagasi* for the diagnosis of canine CVL using an ELISA‐based assay. Our results show that the rLPG3‐ELISA has a high capacity for serum antibody detection in *L. chagasi‐*infected dogs (95.71% sensitivity, with 95% CI = 0.8798–0.9911). This result is better when compared to the results observed from the rapid tests for CVL using the recombinant K39 protein, 87%–93% sensitivity [35, 36], A2 antigen, 87% sensitivity [37], rCatL recombinant protein, 80% sensitivity [15] and DPP‐CVL test, which presents 86% sensitivity with a 78%–92% confidence interval [38]. The latter is even the test recommended by the Brazilian Ministry of Health. It uses DPP to detect antibodies against the chimeric rK28 protein, a result of the K26/K39 fusion of the amastigote antigens (DPP), confirmed by immunoenzymatic assay probing the soluble antigens of promastigotes (ELISA). DPP‐negative animals are considered infection‐free [39]. However, it has been reported that the serological and molecular test diagnoses of CVL in Brazilian endemic areas failed to identify an expressive number of infected animals, revealing that around one out of five seronegative dogs is infected [40].

A comparative analysis between rLPG3‐ELISA and other diagnostic methods widely used in Brazil for CVL, such as IFAT‐CVL alone or in combination with ELISA‐CVL, demonstrated a high level of agreement in the detection of seropositive samples. This similarity in sensitivity, together with the high AUC obtained in the ROC analysis and favourable likelihood ratios, reinforces the potential of rLPG3‐ELISA as a reliable diagnostic tool that may be used as a complementary assay for initial screening, followed by confirmation using methods with higher diagnostic accuracy. However, as these comparisons were conducted using samples without parasitological or molecular confirmation, the observed agreement should be interpreted with caution, as it may reflect concordance between assays rather than true diagnostic accuracy.

However, in our study, a considerable proportion of samples was classified as indeterminate (13.7%). This finding highlights a limitation of the method in clearly distinguishing between positive and negative results. The presence of an indeterminate range may lead to uncertainty in clinical interpretation, hinder decision‐making and ultimately compromise diagnostic reliability. Furthermore, it may require additional testing or repeated analyses, increasing both the time and cost of diagnosis, as well as delaying appropriate clinical or control measures. It is important to note that the serological and molecular methods currently used for the diagnosis of CVL in endemic areas of Brazil fail to identify a significant proportion of infected animals. It is estimated that approximately one in five seronegative dogs may, in fact, be infected. One of the major challenges in CVL serodiagnosis is the detection of asymptomatic dogs, which typically present low antibody titres.

The recombinant antigen rKi‐8.3 has recently been evaluated in ELISA assays, showing promising results, with a sensitivity of 97.1%, which is higher than that observed for rLPG3‐ELISA [41, 42]. Subsequently, this antigen was further optimized by the addition of a tandem repeat tail, combined with an additional ELISA step using a secondary anti‐canine IgG amplifying antibody, aiming to enhance sensitivity, particularly in dogs with low serological scores. The results demonstrated a significant increase in sensitivity without compromising specificity, thereby reducing the likelihood of misdiagnosis [43].

Taken together, these findings highlight the need for further improvement in the sensitivity of the rLPG3‐ELISA. Enhancing its sensitivity may contribute to reducing the frequency of indeterminate and false‐negative results, which are often associated with asymptomatic dogs or those with low antibody levels against *Leishmania infantum chagasi*. Moreover, improving test sensitivity may enhance the identification of asymptomatic dogs with low antibody levels, which is essential for effective epidemiological control of CVL.

The rLPG3‐ELISA demonstrated a specificity of 87.50%, with a 95% confidence interval (CI) ranging from 0.6764 to 0.9734. Specificity is a critical parameter in serological tests, as higher values reduce the occurrence of false‐positive results. This is particularly important in the context of CVL diagnosis, where misdiagnosis can lead to the unnecessary euthanasia of healthy dogs. Therefore, improving specificity directly contributes to better decision‐making in disease management. However, the specificity of the rLPG3‐ELISA was lower compared to other tests reported in the literature, such as the rCatL assay, which showed 95% specificity (95% CI = 0.84–0.99) [15], and the DPP‐CVL, which demonstrated 94% specificity (95% CI = 0.92–0.97) [38]. This discrepancy led us to further investigation into potential cross‐reactivity with other parasitic infections, which may contribute to the reduced specificity observed in the rLPG3‐ELISA. Addressing these factors is crucial for enhancing the diagnostic accuracy of the rLPG3‐ELISA test in future studies.

The high Youden index of 0.8321 further confirms the diagnostic potential of the rLPG3‐ELISA, reinforcing its ability to minimize false positives and false negatives, as indicated by its sensitivity and specificity values [44]. While the specificity was slightly lower than that of other tests, such as the rCatL assay and DPP‐CVL, the overall diagnostic performance of rLPG3‐ELISA remains strong, as evidenced by its ability to discriminate effectively between infected and uninfected cases. However, the presence of indeterminate results and a slightly reduced specificity suggest the need for further refinement in the test's parameters, particularly when addressing potential cross‐reactivity with other parasitic infections.

Significant cross‐reactivity in the rLPG3‐ELISA was observed using sera from animals affected by ehrlichiosis (9 out of the 10 samples tested). The cross‐reaction with serum from dogs affected by *T. cruzi* infection was observed in 8 out of the 39 samples analysed, a lower ratio when compared with ehrlichiosis. Despite the protein conservation observed between *Leishmania* and *Trypanosoma* (showing 68% similarity), it was possible to discriminate between the *L. chagasi* and *T. cruzi* infections in around 80% of the samples analysed with rLPG3‐ELISA.

Although no cross‐reaction occurred between CVL and ehrlichiosis in the tests performed using the LPG antigen [45], when the extract of *L. infantum chagasi* and the A2 antigen were used, a cross‐reaction occurred [46, 47], showing that this is not an unusual problem.

The cross‐reaction observed using the samples with antibodies against *E. canis* in our and other tests may relate to the conservation of the protein in the *L. chagasi* and *E. canis* species. Protein analysis realized in the pBLAST showed 31.52% identity between the LPG3 and Anaplasmataceae chaperone family (MULTISPECIES: molecular chaperone HtpG [*Ehrlichia*]—Sequence ID: WP_045804457.1), with 91% of coverage, a maximum score of 338, and an *E*‐value of 5e−10^6^ (data not shown).

Thus, it is necessary to improve the specificity of the rLPG3 antigen, considering its application under field conditions, where regions endemic for ehrlichiosis may compromise test performance. To address this, refinement of the antigen is essential, either by eliminating regions conserved among other organisms or by selecting immunodominant epitopes that do not share homology with these species. These strategies aim to enhance specificity, but ideally, without compromising test sensitivity.

The mapping of protein‐specific linear epitopes enables the exclusion of regions that share homology with proteins from other pathogens [48]. A major challenge of this approach lies in the fact that immunodominant epitopes that overlap with conserved regions responsible for cross‐reactivity should be removed, consequently reducing test sensitivity using a limited number of epitopes. This is particularly important considering the use of sera from dogs with low antibody titres, potentially leading to inconsistent results [49, 50].

Chemically synthesized linear peptides based on in silico prediction tools can be used as alternative antigens, offering advantages such as low production cost, high specificity and the absence of contaminants derived from bacterial or eukaryotic expression systems commonly used for recombinant protein production [51, 52]. However, these peptides may fail to reproduce conformational epitopes present in the native protein, which can limit antibody recognition in a subset of infected animals.

Another approach that can be used to improve the specificity of diagnostic tests is the use of small immunoreactive peptides obtained from the entire protein. These peptides need to be tested against sera from infected animals by different pathogens, including positive sera to antibodies of interest. Selected peptides that do not react against sera from other pathogens besides the pathogen of interest are eligible to be used in the immunodiagnostic test. Considering this approach, promising peptides identified from rLPG3, for example, can be used alone, in tandem, or combined into chimeric constructs to improve sensitivity while minimizing cross‐reactivity. Studies using combinations of synthetic peptides, either assembled into chimeric constructs or used individually and predicted from *Leishmania* antigens, have demonstrated simultaneous improvements in sensitivity and specificity of serological responses, including in dogs with low antibody titres. These findings suggest that combining epitopes can help balance these diagnostic parameters in ELISA assays [20, 51, 53, 54].

Our research group is now focusing its efforts on the systematic mapping of *L. chagasi*‐specific linear epitopes within the rLPG3 protein, followed by the rational selection and combination of these peptides, either individually or in chimeric constructs, to balance specificity and sensitivity. This strategy has the potential to improve assay reproducibility and reduce cross‐reactivity in the serodiagnosis of CVL using epitopes from rLPG3.

In conclusion, our results demonstrate that the rLPG3 protein has significant potential as an antigen for the serodiagnosis of *L. chagasi* infection in dogs; however, its application in ELISA assays under field conditions still requires improvements in both sensitivity and specificity when compared to currently used antigens. Although the high sensitivity observed reduces the risk of false‐negative results, further refinements are necessary to minimize cross‐reactivity and enhance test specificity and future studies should therefore focus on modifying the antigen to remove epitopes that exhibit cross‐reactivity with antibodies produced against other microorganisms commonly pathogenic to dogs, such as epitopes shared with *E. canis* , to improve diagnostic accuracy.

## Funding

This study was sponsored by the Coordenação de Aperfeiçoamento de Pessoal de Nível Superior—Brasil (CAPES), which funded the executor's doctoral scholarship, Fundação de Amparo à Pesquisa do Estado de Minas Gerais (FAPEMIG—Grant number 9553—FAPEMIG CBB—APQ‐00668‐13), Conselho Nacional de Desenvolvimento Científico e Tecnológico (CNPq) MCTI/CNPq/MEC/Capes—Ação Transversal number 06/2011—Casadinho/Procad and FINEP.

## Conflicts of Interest

The authors declare no conflicts of interest.

## Supporting information


**Figure S1:** Purity analysis of the rLPG3 by capillary electrophoresis. The purity of rLPG3 was evaluated using the purified protein applied in the LabChip Agilent Protein 230 Kit (Agilent 2100 Bioanalyzer). (A) Electrophoresis result after SDS‐PAGE gel visualization. Only one protein band is observed in the rLPG3 line; (B) graphical electrophoresis of the experiment; (C) quantification of the results: rLPG3 was detected with 86.9 kDa, concentrated sample at 1068 ng/μL and a degree of purity 94.7%.

## Data Availability

Research data are not shared.
